# Non-canonical active site architecture of the radical SAM thiamin pyrimidine synthase

**DOI:** 10.1038/ncomms7480

**Published:** 2015-03-27

**Authors:** Michael K. Fenwick, Angad P. Mehta, Yang Zhang, Sameh H. Abdelwahed, Tadhg P. Begley, Steven E. Ealick

**Affiliations:** 1 Department of Chemistry and Chemical Biology, Cornell University, 120 Baker Lab, Ithaca, New York 14853, USA; 2 Department of Chemistry, Texas A&M University, College Station, Texas 77843, USA

## Abstract

Radical *S*-adenosylmethionine (SAM) enzymes use a [4Fe-4S] cluster to generate a 5′-deoxyadenosyl radical. Canonical radical SAM enzymes are characterized by a β-barrel-like fold and SAM anchors to the differentiated iron of the cluster, which is located near the amino terminus and within the β-barrel, through its amino and carboxylate groups. Here we show that ThiC, the thiamin pyrimidine synthase in plants and bacteria, contains a tethered cluster-binding domain at its carboxy terminus that moves in and out of the active site during catalysis. In contrast to canonical radical SAM enzymes, we predict that SAM anchors to an additional active site metal through its amino and carboxylate groups. Superimposition of the catalytic domains of ThiC and glutamate mutase shows that these two enzymes share similar active site architectures, thus providing strong evidence for an evolutionary link between the radical SAM and adenosylcobalamin-dependent enzyme superfamilies.

## Abstract (web summary)

Most radical SAM enzymes use a [4Fe-4S] cluster to generate a 5′-deoxyadenosyl radical. Here the authors show the radical SAM thiamin pyrimidine synthase ThiC comprises an additional active site metal, probably representing an evolutionary link between the radical SAM and adenosylcobalamin-dependent enzyme superfamilies.

## Graphical abstract

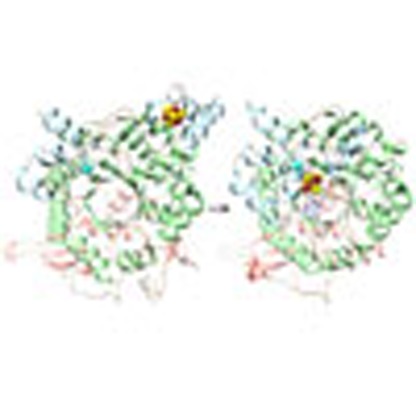

Evidence that *S*-adenosylmethionine (SAM) could be the source of 5′-deoxyadenosyl radicals in enzymes was first discovered through studies of lysine 2,3-aminomutase^1^,^2^. Frey and colleagues^3^,^4^,^5^,^6^ showed that radical formation occurred through homolytic reductive cleavage of a SAM C5′–S bond by using a [4Fe-4S] cluster as the source of an electron. The radical then typically abstracts a substrate hydrogen atom to initiate downstream chemistry. Shortly thereafter, Sofia *et al*.^7^ used bioinformatics to identify over 600 enzymes that constituted the radical SAM superfamily. Today, radical SAM enzymes are known to catalyse a wide range of chemical reactions, including posttranslational modifications of proteins and nucleic acids^8^, with tens of thousands of family members predicted to exist^9^; however, only a few of these have been biochemically and structurally characterized. The radical SAM superfamily is characterized by a (β/α)_8_-barrel or modified β-barrel fold in which the [4Fe-4S] cluster inserts near the C-terminal ends of the β-barrel strands^10^. The CX_3_CX_2_C cluster-binding motif is usually located near the N terminus of the sequence. The three cysteine residues ligate three irons of the cluster and SAM is anchored through its amino and carboxylate groups to the fourth iron^11^. This canonical mode of SAM binding positions SAM for cleavage of the C5′–S bond and generation of the 5′-deoxyadenosyl radical and L-Met (Fig. 1a). 5′-deoxyadenosine (5′-dAdo) is often a product of the reaction, although in some cases SAM serves as a cofactor and is regenerated at the end of the reaction cycle^12^.

Recently, examples of radical SAM enzymes have been identified that differ significantly from the canonical radical SAM superfamily. Variation in the cluster-binding motif, or its location in the sequence, prevents bioinformatics from identifying these as radical SAM enzymes. The radical SAM enzyme PhnJ catalyses cleavage of the C–P bond of phosphonates and has a CX_2_CX_21_CX_5_C motif near the C terminus of the sequence; however, no structure is available and only recently have the three cysteine residues important for cluster binding been identified^13^,^14^. HmdB, involved in the biosynthesis of hydrogenase cofactor, contains a CX_5_CX_2_C cluster-binding motif near the N terminus of the sequence^15^. No structure of the enzyme is available. QueE catalyses the synthesis of 7-carboxy-7-deazaguanine and contains a CX_14_CX_2_C cluster-binding motif. A recent structure shows that QueE contains a (β_6_/α_3_) core and a canonical cluster-binding motif with one extended loop^16^.

ThiC, the enzyme discussed here, catalyses the complex rearrangement of aminoimidazole ribonucleotide (AIR) to 4-amino-5-hydroxymethyl-2-methylpyrimidine phosphate (HMP-P) in the thiamin biosynthetic pathways of bacteria and plants (Fig. 1b)^17^ and is an example of a non-canonical radical SAM enzyme^18^. The CX_2_CX_4_C motif of ThiC varies from the canonical radical SAM motif and is located near the C terminus of the sequence. A mechanism, consistent with detailed labelling studies and the observation of formate and carbon monoxide as additional products, has been proposed^19^. In this mechanism, the 5′-deoxyadenosyl radical initiates the reaction by abstraction of a C5′ hydrogen atom of AIR. The homodimeric structure of *Caulobacter crescentus* ThiC (CcThiC) showed a (β/α)_8_-barrel fold and suggested that the cluster-binding domain, which was disordered, resides near the C terminus and inserts into the active site of an adjacent protomer through domain swapping^18^. The high-resolution structure of *Arabidopsis thaliana* ThiC (AtThiC)^20^ confirmed the earlier results; however, neither ThiC structure contained a [4Fe-4S] cluster nor an ordered cluster-binding motif. We have now used AtThiC and CcThiC to determine a series of structures containing the [4Fe-4S] cluster and various combinations of substrates, products and analogues. These structures define the fold of the ThiC cluster-binding domain and map out the details of the active site.

## Results

### Structure of ThiC with a [4Fe-4S] cluster

ThiC containing the [4Fe-4S] cluster was crystallized anaerobically under various conditions (Table 1). We prepared two different crystal forms of AtThiC, each containing the substrate analogue imidazole ribonucleotide (IRN) and the SAM analogue *S*-adenosylhomocysteine (SAH), which is also a competitive inhibitor of ThiC^21^.

The ThiC structures revealed the details of the [4Fe-4S] cluster and the cluster-binding domain (Fig. 2a,b and Supplementary Fig. 1a), and the binding sites for SAH (Fig. 2c and Supplementary Fig. 1b) and IRN (Fig. 2d and Supplementary Fig. 1c). The cluster-binding domain inserts into the active site of an adjacent catalytic domain through domain swapping as previously predicted^18^. The cluster-binding loop (**C**SM**C**GPKF**C**) is preceded by a 12-residue tether, which connects to the adjacent catalytic domain through a three-helix bundle. The three-helix bundle is located at the dimer interface and is the C-terminal feature of the previously reported ThiC structures^18^,^20^. The cluster-binding loop is followed by a 10-residue α-helix; however, the final 55 residues are not visible in the electron density, even at very high-resolution. Crystals of CcThiC with bound cluster, IRN, SAH and Zn diffracted to lower resolution. The structure is consistent with the AtThiC cluster and cluster-binding domain (Supplementary Fig. 1d), but was not refined.

### SAH-binding site

The mode of SAH binding was unexpected. In the canonical radical SAM enzymes, the SAM amino and carboxylate groups anchor to the differentiated iron in the [4Fe-4S] cluster (Fig. 2e)^22^; however, in ThiC the fourth iron binds to a chloride ion (Supplementary Fig. 2), and the amino and carboxylate groups of SAH anchor to an additional transition metal site (Fig. 2f). The conformation of SAH is also stabilized by van der Waals interactions and through a salt bridge between the carboxylate and absolutely conserved Arg386 (Fig. 2c). SAH forms two hydrogen bonds with Glu489 through its 2′- and 3′-hydroxyl groups. The adenine base is sandwiched between Leu259 and Met572, the N7 atom forms hydrogen bonds with the guanidinium of Arg343 and the amino group forms a hydrogen bond with the backbone carbonyl of Gly230.

### AIR- and IRN-binding sites

The three crystal structures determined for AtThiC with bound IRN show that IRN adopts the same binding mode observed in the previously determined structure of CcThiC that lacked a [4Fe-4S] cluster (Fig. 2d)^18^. Its dominant interaction with ThiC occurs through its phosphate group, which forms hydrogen bonds with the guanidinium group of Arg386, the hydroxyl group of Tyr286, the hydroxyl group of Ser342, the imidazole ring of His322 and the backbone amide groups of Arg343 and Gly344. The ribose hydroxyl groups form hydrogen bonds with the side chains of Asn228 and Glu422, and the N3 atom of the imidazole ring hydrogen bonds with the side chain of Asp383. IRN does not make any direct interactions with SAH.

The high-resolution crystal structures of AtThiC with bound AIR show that AIR makes the same interactions with ThiC as IRN. In addition, the structures reveal the orientation of the imidazole ring of AIR (Fig. 3a) and show that its C5 amino group stacks against the aromatic face of Tyr449.

### 5′-dAdo- and L-Met-binding sites

The high-resolution crystal structure of AtThiC with bound [4Fe-4S] cluster, 5′-dAdo, L-Met and AIR shows that the binding configurations of the SAM cleavage products 5′-dAdo and L-Met are similar to the binding configurations of the 5′-dAdo and homocysteine portions, respectively, of SAH (Fig. 3a and Supplementary Fig. 1e,f). The adenine ring of 5′-dAdo packs between the side chains of Leu259 and Met572. The N7 atom forms hydrogen bonds with the guanidinium of Arg343 and the amino group forms a hydrogen bond with the backbone carbonyl of Gly230. The ribose O2′ and O3′ hydroxyl groups form hydrogen bonds with the side chain of Glu489. The only difference between the conformations of 5′-dAdo and the 5′-dAdo group of SAH is a change in ring pucker of the ribose group, which results in a 3-Å displacement of the C5′ atom.

L-Met chelates to the additional metal site through its amino and carboxylate groups similar to the way the homocysteine portion of SAH chelates to the metal site. The side chain of L-Met is also oriented similarly to the side chain of the homocysteine portion of SAH. The distance between the sulfur atom of L-Met and the Cys573-ligated Fe of the cluster is 3.0 Å.

### EXAFS of AtThiC crystals

In addition to the amino and carboxylate groups of SAH, the metal is coordinated to two absolutely conserved histidine residues (His426 and His490 in AtThiC and His417 and His481 in CcThiC) and two water molecules (Fig. 2f). We used X-ray fluorescence spectroscopy of single crystals (Supplementary Fig. 3) to identify the metal at this site. For AtThiC crystals grown without addition of ZnSO_4_, a strong fluorescence emission peak was observed for Fe and peaks were observed at trace levels for Zn and Ni (Supplementary Fig. 3a). For the crystals grown with addition of ZnSO_4_, strong fluorescence emission peaks were observed for both Fe and Zn, and a trace-level peak was observed for Ni (Supplementary Fig. 3b). The higher level of Zn suggested a bound Zn with the probable site being the metal bound by His426 and His490.

### Multiwavelength anomalous difference Fourier analyses

To provide further evidence for the identity of the additional metal, a multiple wavelength anomalous diffraction experiment was performed. The resulting anomalous difference Fourier maps were very clean and showed strong anomalous scattering signals (Supplementary Fig. 4). Peak heights for a metal at its absorption edge were 60–80 times the root mean square value of the map. The results of the multiwavelength anomalous diffraction experiments using the cluster iron atoms as a reference are shown in Supplementary Table 1 and indicate that in the absence of added ZnSO_4_ the metal is iron, and that when ZnSO_4_ is added during crystallization the iron is displaced by zinc.

### Activity studies of mutant proteins

The prediction that the additional metal site anchors SAM through its amino and carboxylate groups was tested by mutating His417 and His481 in CcThiC individually and together. CcThiC activity was reduced 5-fold for each individual mutation and 15-fold for the double mutation (Supplementary Fig. 5).

### Activity of AtThiC under crystallization conditions

To confirm that the crystallization buffer does not lead to an inhibited form of the enzyme and the failure to properly coordinate SAM due to the presence of chloride, the activity of AtThiC was tested under starting and intermediate crystallization conditions. AtThiC mostly precipitated under final equilibrium crystallization conditions. Both the production of HMP-P and 5′-dAdo were monitored and the results are shown in Supplementary Table 2. Under initial crystallization conditions, AtThiC showed 75% HMP-P formation and 88% 5′-dAdo formation relative to control conditions, and 80% HMP-P formation and 100% 5′-dAdo formation halfway between initial and equilibrium conditions, suggesting that the conformation observed in the crystal form represents a *bona fide* catalytic intermediate.

### Exploration of alternate SAM conformations

A methyl group was added to SAH in the *S*-configuration to create a model of SAM. Although the SAM methionyl group can be pointed generally in the direction of the differentiated iron, it is not long enough to reach the iron and the sulfonium ion is nearly 5 Å from the differentiated iron. In addition, clashes between SAM and the protein atoms occur when the SAM methionyl group is pointed towards the differentiated iron. To more completely explore the alternate SAM conformations, we carried out a Monte Carlo/energy minimization simulation in which the torsion angles of the methionyl portion of SAM were allowed to vary. The lowest energy structure corresponded to the crystal structure. None of the additional low-energy structures corresponded to a conformation in which both the SAM amino and carboxylate groups were anchored to the differentiated iron.

## Discussion

The high-resolution structure of AtThiC co-crystallized with SAH or with 5′-dAdo and L-Met showed a consistent arrangement with atoms of SAH closely aligned with the corresponding atoms in 5′-dAdo and L-Met (Figs 2f and 3a). The L-Met amino and carboxylate groups chelate to the additional metal ion and the adenosyl moiety superimposes closely with that of SAH. We also determined the structure of AtThiC co-crystallized with AIR and SAH (Supplementary Fig. 1g,h), and showed that AIR and IRN superimpose closely. The ensemble of five high-resolution AtThiC structures containing various combinations of AIR, IRN, SAH, L-Met and 5′-dAdo show an unexpected, but self-consistent, pattern of substrate, product and analogue-binding geometry.

Although the identity of the additional metal *in vivo* is not known, it was first observed in our original structure of CcThiC and determined to be zinc by EXAFS^18^. The metal was modelled as cobalt in the original structure of AtThiC, because CoCl_2_ was present in the crystallization conditions; however, the identity of the metal was not confirmed^20^. Formation of the [4Fe-4S] cluster in ThiC requires elevated concentrations of iron in the culture medium, and for crystals prepared this way the metal was assigned as iron. Zinc when added to AtThiC at a 1:1 molar ratio during crystallization largely displaces iron at the additional metal site.

A comparison of the [4Fe-4S] cluster with bound SAM from canonical radical SAM enzymes and AtThiC led to another unexpected observation (Fig. 2e,f). In the canonical radical SAM enzymes, the conformation of SAM places the SAM sulfur atom near the differentiated iron with an approximately linear Fe…S-C5′ arrangement as required for cleavage of the C5′–S bond and formation of the 5′-deoxyadenosyl radical^11^,^23^. Using deposited high- or very-high-resolution structures of radical SAM structures with bound SAM (PDB IDs 1OLT, 2A5H, 2FB2, 3CB8, 3IIZ, 3RFA, 3T7V, 4FHD, 4K39 and 4M7T), the Fe…S distance ranges from 3.1 to 3.6 Å and the Fe…S-C angle ranges from 139° to 161°. In ThiC, the fourth iron of the cluster bonds to chloride. In the high-resolution crystal structures of AtThiC containing SAH, the average Fe…S distance from the iron covalently bound to Cys573 is 3.5 Å (range 3.3–3.7 Å) with an average Fe…S-C angle of 165° (range 162°–169°). In the structures containing L-Met, the Fe…S distance is ~3.0 Å from this iron.

The structure of ThiC with bound AIR and SAH, and the demonstration of consistent binding geometries among our collection of structures allowed us to readily generate a model of the ThiC/AIR/SAM complex by adding a methyl group in the *S*-configuration to the sulfur atom of SAH (Fig. 3b). Adding the methyl group in the *R*-configuration resulted in steric clashes. In the model of ThiC/AIR/SAM, all SAH atoms were kept fixed and no ThiC conformational changes were required. The model shows that SAM is poised for C5′–S bond cleavage after which the C5′ position of the 5′-deoxyadenosyl radical would be ideally positioned to abstract the C5′-proS hydrogen atom of AIR (C5′...proS H distance 3.2 Å and C5′...proR H distance 4.2 Å).

The unexpected active site geometry of SAM, inferred from SAH or 5′-dAdo and L-Met, raises the possibility that our structures represent inhibitory states of the enzyme or crystallization artefacts, and that in the active form of ThiC SAM would be anchored by the differentiated iron just as it is in the canonical radical SAM superfamily members. To test this possibility, we superimposed a representative [4Fe-4S] cluster and its attached SAM molecule (taken from HydE^22^) onto the ThiC cluster in the three possible orientations (corresponding to 120° rotations about the cluster diagonal containing the differentiated iron) that would allow the SAM amino and carboxylate groups to anchor to the differentiated iron of the ThiC cluster. In each case, the superimposition showed severe clashes, with SAM interpenetrating protein side chains and no possibility of alleviating the clashes (Supplementary Fig. 6). Furthermore, none of the possibilities would place the 5′-deoxyadenosyl radical close enough to the substrate AIR for abstraction of the 5′-hydrogen atom as required by the proposed mechanism^19^. Attempts to reposition the cluster through conformational changes in the tethered region also failed to achieve the canonical radical SAM active site architecture. Finally, studies using conformational searching showed that the lowest energy conformation for SAM bound to the AtThiC active site corresponds to the crystal structure with SAH. All of these observations support a new, non-canonical active site architecture for AtThiC.

The original structure of CcThiC showed that its most closely related structural homologues were adenosylcobalamin (AdoCbl)-dependent enzymes^18^. This observation is consistent with the proposal based on functional and structural considerations that radical SAM enzymes and AdoCbl-dependent enzymes might have an evolutionary relationship^24^,^25^. Drennan and colleagues^10^ proposed a model for 5′-dAdo binding in ThiC, using a superimposition of the structures of ThiC lacking the [4Fe-4S] cluster^18^ and *Clostridium cochlearium* glutamate mutase complexed with AdoCbl and L-glutamate^26^. ThiC and glutamate mutase have structurally homologous catalytic domains containing the substrate (AIR or L-glutamate) binding site. ThiC contains a tethered [4Fe-4S] cluster-binding domain, while glutamate mutase contains a separate chain for binding the AdoCbl cofactor (Fig. 4a). The model predicted that a conserved glutamate side chain (Glu489 in AtThiC) would hydrogen bond to the 2′- and 3′-hydroxyl groups of 5′-dAdo. The model also proposed that a hydrophobic residue (Leu259 in AtThiC) would pack against the adenine ring. Superimposition of the ThiC structures from our crystallographic studies with glutamate mutase (PDB ID 1I9C) confirmed these predictions (Fig. 4b,c). In addition to Glu489 and Leu259, Gly230, Leu493 and Pro494 are also structurally and functionally conserved in glutamate mutase. Furthermore, the ThiC AIR-binding site and the glutamate mutase L-Glu-binding site overlap spatially, and the cobalamin cobalt is near the iron in ThiC that is predicted to interact with the sulfonium ion of SAM (Fig. 4d). This comparison not only supports the non-canonical active site architecture of ThiC but also provides strong evidence for an evolutionary link between the radical SAM and AdoCbl-dependent enzyme superfamilies.

One structure of CcThiC and one structure of AtThiC stand apart and provide insight into conformational changes occurring in ThiC. In the holo CcThiC structure, the entire C-terminal cluster-binding domain (Supplementary Fig. 7a) and [4Fe-4S] cluster (Supplementary Fig. 7b) are clearly defined; however, the cluster-binding domain extends away from the active site and the cluster itself is ~25 Å from its active site location (Fig. 5a,b). The structure of AtThiC co-crystallized with only IRN shows well-defined density for the catalytic domain and clear density for IRN (Supplementary Fig. 7c); however, the density beyond Glu557, which includes the cluster-binding domain, is absent, indicating that the cluster-binding domain is disordered. In contrast, every structure containing SAH or L-Met and 5′-dAdo shows a well-ordered cluster-binding domain. In addition to the disordered cluster-binding domain, the AtThiC/IRN structure shows conformational changes for residues 227–247, 257–277 and 487–500 (Fig. 6). These protein regions are involved in contacts between the catalytic domain and the cluster-binding domain, and contain residues Leu259, Glu489 and Leu493, which make contacts with SAM in the ThiC/AIR/SAM model.

The ensemble of X-ray structures and the ThiC/substrate model suggest the substrate AIR binds first (similar to IRN in Fig. 2d) and is oriented through hydrogen bonds between the phosphate and the side chains of Tyr286, His322, Ser342 and Arg386, and backbone amides of Arg343 and Gly344. The phosphate group is located at the N-terminal end of an α-helix (343–356) and further stabilized by the helix dipole. Hydrogen bonds also form between the AIR ribosyl hydroxyl groups and Asn228 and Glu422. The imidazole N3 atom is within hydrogen-bonding distance from the Asp383 side chain. The large number of interactions with phosphate and the similarity of the phosphate site to that of HMP-P in our previously reported structure^18^ suggest that it acts as an anchor throughout the catalytic cycle. SAM binds second (similar to SAH in Fig. 2c) with its amino and carboxylate groups anchored to the additional metal ion, its ribosyl hydroxyl groups hydrogen bonded to Glu489 and the adenine ring sandwiched between Leu259 and Met572, which comes from the cluster-binding loop. This geometry positions the SAM 5′-carbon atom 3.2 Å from the AIR 5′-proS hydrogen atom; however, no other direct contacts with AIR are observed. This is in contrast to canonical radical SAM enzymes, where interactions between SAM and the substrate are often extensive and orient reactants for initiation of radical chemistry. SAM binding induces three conformational changes, which facilitate docking of the cluster-binding domain to the active site (Fig. 6). Docking of the catalytic and cluster-binding domains positions the iron bonded to Cys573 near the SAM sulfonium ion. Subsequent C5′–S bond cleavage results in 5′-deoxyadenosyl radical formation and abstraction of the AIR 5′-proS hydrogen atom. This order of binding, where AIR binds first and SAM stabilizes interactions with the cluster-binding domain, controls initiation of radical chemistry and most probably prevents uncoupled turnover of SAM *in vivo*.

In summary, the ensemble of ThiC structures reported here demonstrates several unprecedented features of ThiC *vis a vis* the radical SAM enzyme superfamily. (i) The binding mode of SAM (inferred from structures with SAH or 5′-dAdo and L-Met) is unique. (ii) The amino and carboxylate groups of SAH are not anchored by the [4Fe-4S] cluster, but instead are anchored by an additional metal ion. (iii) The sulfur atom of SAH is in close proximity to an iron of the [4Fe-4S] cluster, but unlike canonical radical SAM enzymes it is not the differentiated iron but is the iron ligated to Cys573. (iv) Comparison of the structures of ThiC with a [4Fe-4S] cluster and glutamate mutase with AdoCbl provides further evidence for an evolutionary relationship between the two superfamilies. (v) The positioning of the [4Fe-4S] cluster in the active site, along with two structures lacking SAH or L-Met and 5′-dAdo, suggests that the tethered ThiC cluster-binding domain moves into the ThiC active site to initiate catalysis and out of the active site to release products.

## Methods

### Anaerobic production and crystallization of AtThiC

Previous studies reported that overexpression of full-length AtThiC containing 644 residues results in insoluble protein, whereas overexpression of ΔN71-AtThiC with residues 1–71 removed results in soluble protein with high yield^20^,^27^. In the present study, *ΔN71-AtThiC* was cloned into a modified pET-28 plasmid that encodes the following hexahistidine-tagged protein product: NH_2_- MGSDKIHHHHHHSSGENLYFQGHMK_72_…K_644_-COOH.

This plasmid along with plasmid pSuf containing the *Escherichia coli suf* operon were transformed into *E. coli* NiCo21(DE3) cells (New England Biolabs)^28^,^29^,^30^. Large-scale cultures were grown in baffled shaker flasks containing 1.5 l of Luria–Bertani (LB) medium, 0.051 g chloramphenicol and 0.06 g kanamycin. The flasks were shaken at 180 r.p.m. and 37 °C until an optical density at 600 nm (OD_600_) of 0.55–0.6 was reached and then moved to a 4 °C cold room. About 2.5 h later, 200 mg Fe(NH_4_)_2_(SO_4_)_2_·6H_2_O, 200 mg L-Cys and 36 mg isopropyl β-D-1-thiogalactopyranoside (IPTG) were added per flask and the flasks were shaken at 90 r.p.m. and 15 °C for about 20 h. The flasks were then moved to the cold room for about 3 h before being pelleted by centrifugation.

Cell pellets were brought into a PVC anaerobic chamber (Coy Laboratory Products) and re-suspended in lysis buffer (100 mM Tris, pH 7.6, 5 mM dithiothreitol (DTT), 0.4 mg ml^−1^ lysozyme, 1.9 kU benzonase). The cells were further lysed via sonication and the lysate was moved out of the glove box for centrifugation at 60,000*g* and 4 °C for 20 min. The spun lysate was returned to the glove box and the supernatant was subjected to immobilized nickel affinity chromatography employing wash (100 mM Tris, 300 mM NaCl, 20 mM imidazole and 2 mM DTT, pH 7.6) and elution (100 mM Tris, 300 mM NaCl, 250 mM imidazole, pH 7.7) buffers. Eluted protein was buffer exchanged into 25 mM Tris and 150 mM NaCl, using a Bio-Rad Econo-Pac 10DG desalting column, and incubated for 5–14 h in the glove box with tobacco-etch virus protease for cleavage of the hexa-His tag. Subtractive immobilized nickel affinity chromatography was then performed, after which time the isolated protein was buffer exchanged into 5 mM HEPES buffer and 22 mM NaCl, pH 7.0. The protein used for crystallization was previously shown to be active^20^.

Crystals were grown in the anaerobic chamber at room temperature using hanging-drop vapour diffusion, with drops containing a 1:1 ratio of protein to reservoir solution. The concentration of AtThiC was ~17 mg ml^−1^. The final concentrations of ligands co-crystallized with AtThiC were as follows: AIR, 3 mM; IRN, 3 mM; L-Met, 7 mM; 5′-dAdo, 4 mM; SAH (in dimethylsulfoxide), 5 mM; SAM (Sigma or Cayman), 5–7 mM; and ZnSO_4_, 0.23 mM. For the AtThiC crystals having space group P3_2_21, typical reservoir solutions were 100 mM Na acetate pH 5.3–5.6 and 1–9% (v/v) 1,4 butanediol. For the AtThiC crystals having space group C2, the reservoir solution was 100 mM imidazole pH 6.6, 200 mM NaCl and 20% (w/v) polyethylene glycol (PEG) 8000. Crystals were harvested and cryocooled in liquid N_2_ using 32% (v/v) 1,4-butanediol and 38% (w/v) PEG4000 as cryoprotectants, respectively.

### Anaerobic production and crystallization of CcThiC

Full-length *CcThiC* was cloned into the aforementioned modified pET28 vector to give the expressed product: NH_2_- MGSDKIHHHHHHSSGENLYFQGHM_1_…E_612_-COOH.

This plasmid and plasmid pDB1282 (ref. 31) were transformed into *E. coli* B834 (DE3) cells. Large-scale cultures were grown in 1.8 l of minimal medium (21 g M9 salts, 7.5 g glucose, 0.45 g MgSO_4_ and 28 mg CaCl_2_·2H_2_O) per flask supplemented with 0.21 g L-Met, 0.19 g ampicillin and 0.075 g kanamycin. The flasks were shaken at 180 r.p.m. and 37 °C until an OD_600_ of 0.20–0.25 was reached, at which point ~140 mg Fe(NH_4_)_2_(SO_4_)_2_·6H_2_O, 140 mg L-Cys and 5 g L-(+)-arabinose were added per flask. The flasks were then shaken at 50 r.p.m. and 37 °C until the OD_600_ reached about 0.65 and then cultures were moved to a cold room set at 4 °C. After 4–5 h, 6.4 mg of IPTG were added per flask and the flasks were shaken at 50 r.p.m. and 15 °C for about 20 h. Afterwards, the cultures were moved to the cold room for about four hours prior to centrifugation.

The procedures for anaerobic preparation of CcThiC including cleavage of the hexa-His tag and crystallization were identical to those followed for AtThiC. To crystallize holo CcThiC with the [4Fe-4S] cluster remotely docked at His347, protein samples of ~22 mg ml^−1^ CcThiC were combined with typical reservoir solutions containing 140 mM HEPES pH 9.2 and 7.5% (w/v) PEG8000; the cryoprotectant was 40% (w/v) PEG4000. For co-crystallization of CcThiC, IRN, SAH and Zn, protein samples containing ~12 mg ml^−1^ CcThiC were supplemented with 5 mM IRN (prepared previously^18^ in NaH_2_PO_4_ and NaCl), 20 mM HEPES pH 7.8, 5 mM SAH, 1.7% (v/v) dimethylsulfoxide, 0.18 mM ZnSO_4_ and 0.9 mM HEPES, pH 6.9. Typical reservoir solutions consisted of 0.79–0.81 M sodium citrate and between 100 and 200 mM imidazole pH 7.7–8.6; 1.6 M sodium citrate was used for cryoprotection.

### X-ray diffraction data for CcThiC and AtThiC

All X-ray diffraction data were measured at 100 K at beamline 24-ID-C of the Advanced Photon Source, Argonne National Laboratory, using a PILATUS 6M-F detector and a wavelength of *λ*=0.979 Å. Diffraction images were recorded in shutterless mode at a rate of 1 degree per second. Typical rotation ranges were 80°–120° for space group P3_2_21 and 120°–155° for space group C2. Data were integrated and scaled by using HKL2000 (ref. 32).

### Structure determination and refinement of CcThiC and AtThiC

The structures of CcThiC and AtThiC complexed with various ligands were determined by either difference Fourier synthesis or molecular replacement using the published structures PDB ID 4N7Q (AtThiC)^20^ or PDB ID 3EPN (CcThiC)^18^ as the starting models. The structures were refined iteratively using programmes Phenix^33^ and COOT^34^, and validated using MolProbity^35^. The final refinement statistics are summarized in Table 1. Supplementary Fig. 1, showing the quality of the electron density maps and confirming the assignments of the ligands, was prepared using PyMOL^36^.

During the refinement of the very high-resolution structures, the occupancy for the [4Fe-4S] cluster was found to be ~70%. Electron density near the differentiated iron of the cluster was much too large to be explained by a water molecule (average peak height of 20 times the root mean square value of the map). Assigning this peak as chloride resulted in an average B-factor after refinement of ~18 Å^2^ (average protein atom B-factor around 15 Å^2^). The occupancies of the cluster atoms and chloride ion were constrained to the same value during refinement. The 1.25-Å resolution structure of AtThiC with SAH and AIR showed two conformations for SAH. Although atoms of the adenine ring and the aminocarboxypropyl group were the same, the ribosyl group pucker was different (C2′-*exo*, C3′-*endo* for one conformation and C1′-*exo* for the other) causing changes mainly in the positions of O2′, C3′, O3′, C4′, C5′, Cγ and S. In both conformations the SAH amino and carboxyl groups chelate to the additional iron.

A Ramachandran analysis shows that for all structures all torsion angles are in the allowed or favoured regions, except for Ser236, which has clear electron density. The Ser236 side chain forms a strong hydrogen bond with the side chain of Glu241 (2.5 Å) and is located at the end of a loop that interacts with the cluster-binding domain.

### X-ray fluorescence spectra for AtThiC crystals

Crystal structures of AtThiC with a [4Fe-4S] cluster showed a metal bound by His426 and His490. This metal presumably originated from iron in the culture medium, which included LB medium and Fe(NH_4_)_2_(SO_4_)_2_·6H_2_O during protein induction, or from ZnSO_4_ added during crystallization. To determine the identity of the metal, X-ray fluorescence spectra were recorded at beamline 24-ID-C of the Advanced Photon Source (Argonne National Lab), which is equipped with an Amptek XR-100SDD silicon drift detector. Spectra were obtained for AtThiC crystals and associated cryo-solutions for the energy range 500–16,000 eV. Spectra were recorded for co-crystals of AtThiC, AIR and SAM, and co-crystals of AtThiC, AIR, SAM and Zn. For the latter, ZnSO_4_ was added to a final concentration of 0.22 mM before crystallization (corresponding to a 1:1 molar ratio with AtThiC); Zn was not included in the cryoprotectant. Spectra were also recorded for co-crystals of AtThiC, IRN and SAH, with or without added ZnSO_4_.

### Multiwavelength anomalous difference Fourier analyses

Four data sets were collected for each of two crystals, one without and one with added ZnSO_4_. Wavelengths were chosen at (7,132 eV) and below (7,100 eV) the Fe absorption edge and at (9,673 eV) and below (9,650 eV) the Zn absorption edge. Anomalous diffraction data sets were processed using HKL2000 (ref. 32) and analysed using COOT to determine relative anomalous difference Fourier peak heights^34^.

### DNA primers for cloning and mutagenesis

The primers used for cloning AtThiC are 5′- aaaacatatgaaacacaccattgatcctt -3′ (forward) and 5′- aaaactcgagttatttctgagcagcttt -3′ (reverse); the recognition sequences for NdeI and XhoI are underlined. The primers used for cloning CcThiC are 5′- gggtagcatatgaatatccagagcaccatcaagg -3′ (forward) and 5′- cccctaggatcctcactcggtcttcagatagatc -3′ (reverse); the recognition sequences for NdeI and BamHI are underlined. The primers used for making the mutant CcThiC H417A are 5′- gatgatcgaagggccgggcGCGgtggccatgcacaagatcaagg -3′ (forward) and 5′- ccttgatcttgtgcatggccacCGCgcccggcccttcgatcatc -3′ (reverse); capitalized nucleotides mark the site of mutation. The primers used for making CcThiC H481A are 5′- ctacgtcacgcccaaggagGCGctgggcctgccggaccgcg -3′ (forward) and 5′- cgcggtccggcaggcccagCGCctccttgggcgtgacgtag -3′ (reverse).

### Production of CcThiC mutant proteins

The mutant plasmids were co-transformed with pDB1282 in *E. coli* B834 (DE3). ThiC variants were co-expressed in the presence of a pDB1282 plasmid encoding the *Isc* operon for *in vivo* biosynthesis of [4Fe-4S] in *E. coli* B834 (DE3). An overnight 15-ml culture was grown in LB media in the presence of kanamycin (40 mg l^−1^) and ampicillin (100 mg l^−1^). This was then added to 1.8 l minimal media (containing M9 minimal salts 1 × , 27 ml 50% (w/v) glucose, 7 ml 1 M MgSO_4_, 200 μl 1 M CaCl_2_, 200 mg L-Met, 72 μg kanamycin and 180 μg ampicillin). The cultures were incubated at 37 °C with shaking (180 r.p.m.) until the OD_600_ reached 0.2 to 0.25. Next, 5 g of L-(+)-arabinose, 120 mg of Fe(NH_4_)_2_(SO_4_)_2_·6H_2_O and 120 mg of L-Cys were added. The cultures were incubated at 37 °C with shaking (50 r.p.m.) until the OD_600_ reached 0.65 to 0.7. The cultures were then incubated at 4 °C without shaking for 3–4 h. This was followed by induction of the culture with 15 μM IPTG and incubation at 15 °C with shaking (50 r.p.m.) for 18–20 h. The cultures were then incubated at 4 °C for 3 h without shaking. The cells were then harvested and stored in liquid nitrogen overnight before enzyme purification. For enzyme purification, the cell pellets were thawed at room temperature in an anaerobic chamber and suspended in lysis buffer (100 mM Tris-HCl, pH 7.5) in the presence of 2 mM DTT, lysozyme (0.2 mg ml^−1^) and benzonase (100 units). This mixture was then cooled in an ice-bath for 2 h. The suspension of cells was sonicated and centrifuged, to give the cell-free extract. The enzyme was purified using standard Ni-NTA chromatography. The column was first incubated with the lysis buffer. The cell-free extract was passed over the column, which was then washed with 8–9 column volumes of wash buffer (100 mM Tris-HCl, 300 mM NaCl, 20 mM imidazole, 2 mM DTT, pH 7.5). The enzyme was eluted using 100 mM Tris-HCl, 300 mM NaCl, 250 mM imidazole and 2 mM DTT, pH 7.5. The purified enzyme was buffer exchanged into 100 mM potassium phosphate, 30% (v/v) glycerol, 2 mM DTT, pH 7.5, using an Econo-Pac 10DG desalting column (Bio-Rad) and the purified enzyme was stored in liquid nitrogen. The proteins as isolated bind to a [4Fe-4S] cluster.

### Activity studies of mutant proteins

The CcThiC mutant (200 μM) was incubated with 10 mM dithionite, 5 mM AIR and 7 mM SAM at room temperature for 90 min. The protein was filtered out using 10-kDa cutoff filters and the small molecule pool was analysed by HPLC. An Agilent 1260 HPLC was used for detection of the products by ultraviolet absorption at 254 nm. An SPLC-18 column (3.0 × 150 mm, 3 μm, Supelcosil, 25 cm × 10 mm, 5 μm) was used for the HPLC analysis. These experiments were performed twice for each CcThiC mutant.

Chromatography conditions were as follows: (A) water, (B) 100 mM potassium phosphate, pH 6.6, (C) methanol. Flow rate: 2 ml min^−1^. The following gradients were used: 0 min—100% B; 7 min—10% A:90% B; 12 min—25% A:60% B: 15%C; 17 min—25% A: 10% B: 65% C; 19 min—100% B; 29 min—100%A.

### Activity of AtThiC under crystallization conditions

Activity assays were performed at the crystallization conditions to test whether the crystallization solution, in particular chloride, inhibits the AtThiC-catalysed conversion of AIR and SAM to 5′-dAdo and HMP-P. Co-crystals of AtThiC and SAH were grown via vapour diffusion from drops formed from a 1:1 ratio of protein solution (230 μM AtThiC, 5 mM SAH, 5 mM HEPES, 22 mM NaCl, pH 7.0) and reservoir solution (1–9% (v/v) 1,4-butanediol and 100 mM Na acetate, pH 5.3–5.6). Drop equilibration causes protein precipitation and crystallization, and precipitation occurs more readily at the lower end of the pH range. To reduce the amount of precipitation for the activity assays, reduced protein concentrations (100 μM) were required and assays were performed for initial and midway-equilibrated drop solutions at pH 5.6.

Activity was measured for the following three solutions: (i) the initial crystallization drop solution (2.5 mM HEPES, 11 mM NaCl, 1% (v/v) 1,4-butanediol and 50 mM Na acetate, pH 5.6). This corresponds to a 1:1 mixture of the protein and reservoir solutions. The chloride concentration is 17.3 mM, which derives from 11 mM NaCl plus the amount of HCl required to titrate 50 mM Na acetate to pH 5.6 (6.3 mM). (ii) A midway-equilibrated drop solution (3.75 mM HEPES, 16.5 mM NaCl, 1.5% (v/v) 1,4-butanediol and 75 mM Na acetate, pH 5.6). The chloride concentration is 26 mM. (iii) The standard solution used for activity assays (100 mM K_2_HPO_4_ pH 7.5 and 30% (v/v) glycerol).

Activity assays were performed within a Coy anaerobic chamber. Frozen aliquots of AtThiC in 100 mM K_2_HPO_4_ pH 7.5 and 30% (v/v) glycerol were thawed and desalted into each of the above solutions, using BioRad spin desalting columns. AIR (5 mM), SAM (7 mM) and dithionite (20 mM) were then added and reactions were incubated at room temperature for 90 min. The reactions were then quenched by heating followed by centrifugation and the supernatant was passed through a 10-kDa cutoff filter. This was analysed by HPLC for 5′-dAdo and HMP-P formation. Amounts of 5′-dAdo and HMP-P were normalized to protein concentrations. These experiments were performed once for each condition.

### Exploration of alternate SAM conformations

A conformational search was performed to explore the possibility of alternate conformations of SAM in which SAM is bound to the differentiated iron of the [4Fe-4S] cluster through both its amino and carboxylate groups. The calculations were carried out using the programme Macromodel (Schrödinger, Inc.) with the OPLS 2005 force field^37^. SAM was modelled by adding a methyl group to SAH in the crystal structure of AtThiC with bound AIR, SAH and Fe. The Monte Carlo Mulitple Minima programme was used to randomly vary the torsion angles of the methionyl portion of SAM, while the ribose and adenine portions were held fixed. Full residues and ligands having at least one atom within 5 Å of the methionyl moiety of SAM were included in the model. A total of 100,000 conformations were sampled and an energy range of 50 kcal mol^−1^ was used to save an extensive number of candidate structures above the lowest energy structure, which corresponded to the crystal structure. Conformations were energy minimized with the energy gradient threshold set to 1 kJ mol^−1^ Å^−1^.

### Figure preparation

**Figure 1:**
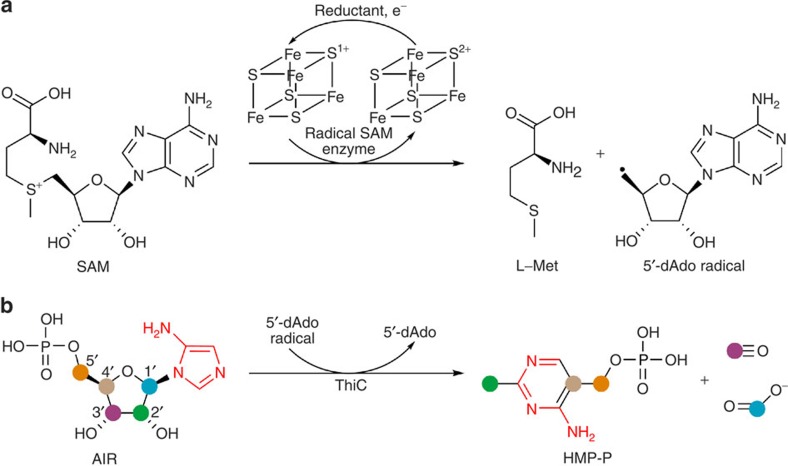
Radical SAM reactions and ThiC. (**a**) Canonical radical SAM reaction scheme. Canonical radical SAM enzymes use a [4Fe-4S] cluster to convert SAM into L-Met and a 5′-deoxyadenosyl radical. The reduced [4Fe-4S] cluster delivers an electron to SAM, resulting in homolytic cleavage of the C5′–S bond. (**b**) Complex rearrangement reaction catalysed by ThiC. ThiC converts AIR to HMP-P, formate and carbon monoxide. The coloured circles indicate the origin of carbon atoms. In a recently proposed mechanism, the reaction commences when the 5′-deoxyadenosyl radical abstracts a C5′ hydrogen atom of AIR^19^.

**Figure 2:**
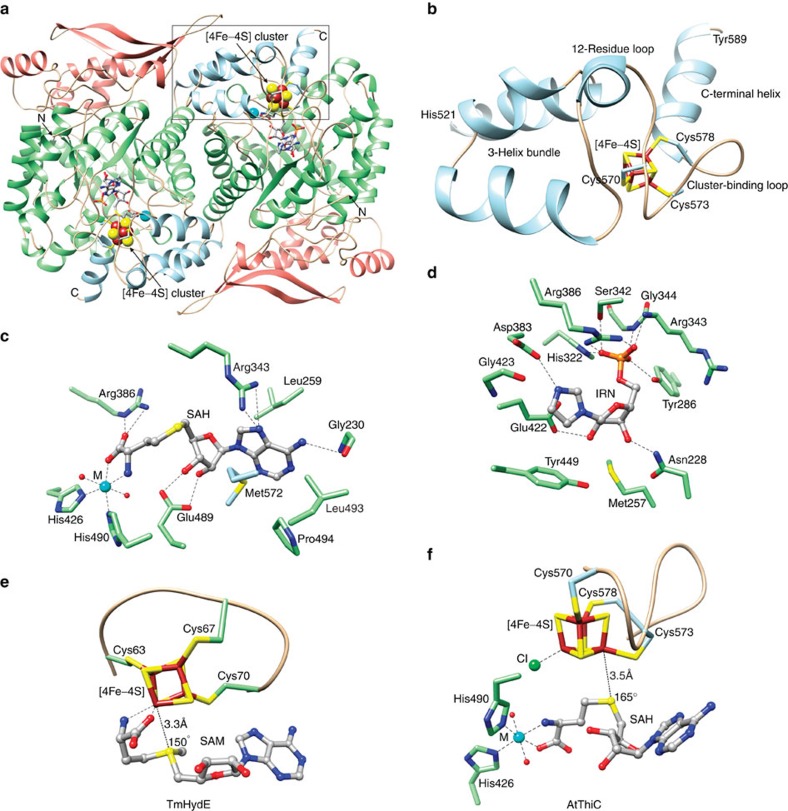
Structure of the [4Fe-4S] cluster-binding domain and active site in AtThiC. (**a**) Structure of AtThiC with [4Fe-4S] cluster. AtThiC is a homodimer with each monomer having an N-terminal domain (salmon), a (β/α)_8_ core domain (green) and a C-terminal cluster-binding domain that inserts into the active site of a twofold related monomer (blue). (**b**) AtThiC cluster-binding domain. The [4Fe-4S] cluster-binding domain is anchored to the adjacent catalytic domain through a three helix bundle. The CX_2_CX_4_C cluster-binding motif is preceded by a 12-residue loop and followed by a 10-residue α-helix. (**c**) SAH-binding site from the ThiC/SAH/IRN complex. (**d**) IRN-binding site from the ThiC/SAH/IRN complex. Hydrogen bonds for **c** and **d** are shown as dashed lines. (**e**) Mode of SAM binding in canonical radical SAM enzymes. SAM anchors to the differentiated iron of the [4Fe-4S] cluster via its amino and carboxylate groups. The C5′-S bond is positioned such that the SAM sulfonium ion is typically 3.1–3.6 Å from the differentiated iron of the [4Fe-4S] cluster with a nearly linear arrangement of Fe…S-C5′ (typically about 150°). (**f**) Mode of SAH binding in ThiC. SAH does not anchor to the [4Fe-4S] cluster, but instead anchors to a secondary metal via its amino and carboxylate groups. The differentiated iron binds to chloride in our structures. The SAH sulfur atom is 3.5 Å from iron and the Fe…S-C5′ angle is 165°.

**Figure 3:**
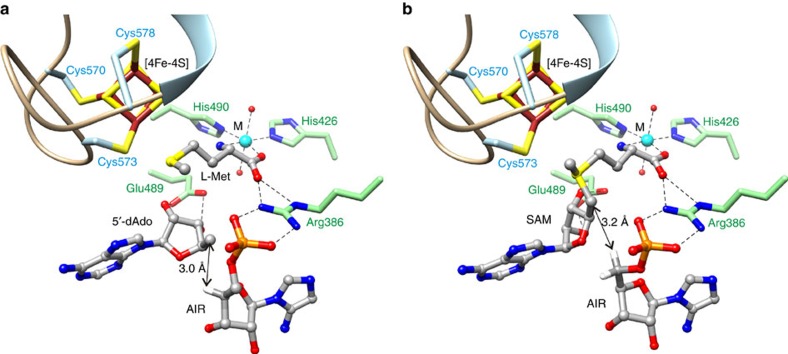
Structures of the AtThiC product complex and model of the ThiC substrate complex. (**a**) X-ray structure of AtThiC with AIR and bound products 5′-dAdo and L-Met. The structures of 5′-dAdo and L-Met are consistent with cleavage of SAM having its carboxylate and amino groups anchored to the additional metal. After cleavage, the carboxylate and amino groups of L-Met remain anchored to the metal. (**b**) Model for the complex of AIR and SAM bound to the AtThiC active site. The model was generated by adding a methyl group to SAH, to give the *S*-enantiomer of SAM (note: the inactive *R*-enantiomer could not be generated without creating steric clashes). In this model, the distance between the C5′ atom of SAM and the proS hydrogen atom of AIR is 3.2 Å. After S–C5′ bond cleavage, the orientation of the 5′-deoxyadenosyl radical would be favourable for abstraction of the AIR C5′-proS hydrogen atom.

**Figure 4:**
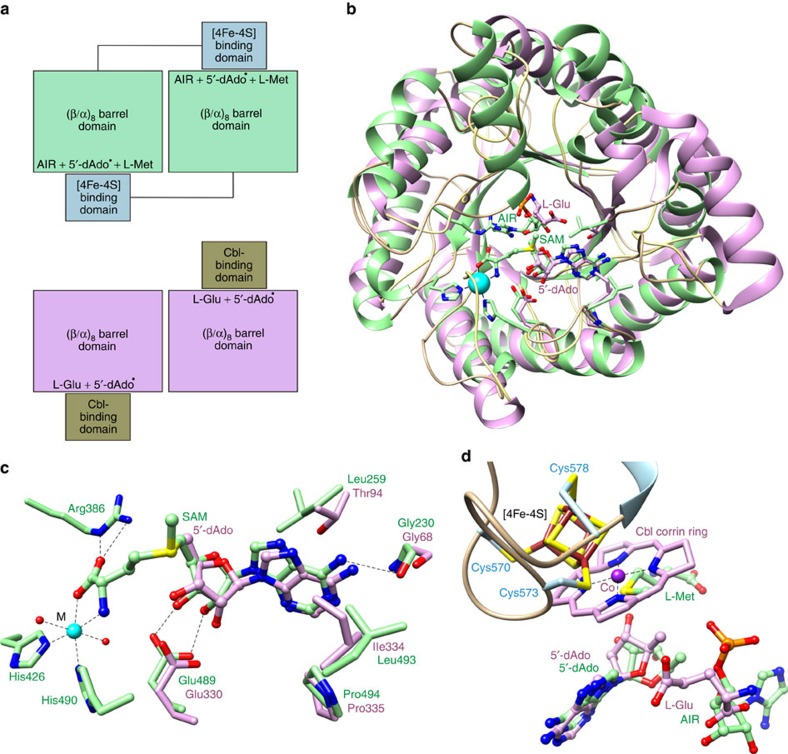
Comparison of ThiC- and AdoCbl-dependent glutamate mutase. (**a**) Organization of ThiC and glutamate mutase dimers. The enzymes have closely related (β/α)_8_ folds and dimer interfaces. ThiC contains a tethered cluster-binding domain that inserts into the twofold related catalytic domain through domain swapping. The AdoCbl-binding domain is a separate chain in glutamate mutase. (**b**) Superimposition of the catalytic domains of AtThiC and glutamate mutase showing the locations of the substrates (AIR or L-glutamate) and SAM (modelled from SAH), and the 5′-deoxyadenosyl moiety of AdoCbl. The ThiC cluster-binding domain and the glutamate mutase AdoCbl-binding domain, which have very different folds, were omitted for clarity. (**c**) Close-up view of the conserved binding site for the 5′-deoxyadenosyl moieties of ThiC and glutamate mutase. Functionally conserved residues that contact the 5′-deoxyadenosyl moieties are shown. (**d**) Location of the metal centres, 5′-dAdo and substrates after superimposition of the ThiC and glutamate mutase catalytic domains. The Cys573-ligated iron that binds the sulfonium atom of SAM and the cobalt that binds the 5′-deoxyadenosyl moiety of glutamate mutase are ~2 Å apart, consistent with the insertion of the SAM sulfonium ion.

**Figure 5:**
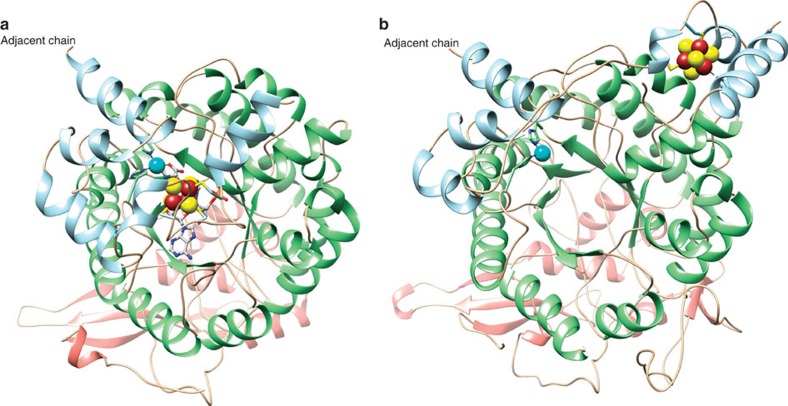
Movement of the ThiC cluster and cluster-binding domain. (**a**) AtThiC protomer structure with bound [4Fe-4S] cluster, SAH, AIR and Fe. (**b**) Holo CcThiC protomer structure. In the absence of substrates, the [4Fe-4S] cluster is located at a remote site in the twofold related protomer, 25 Å away from the active site location of the cluster. The metal (cyan) and conserved histidine residues are included for reference.

**Figure 6:**
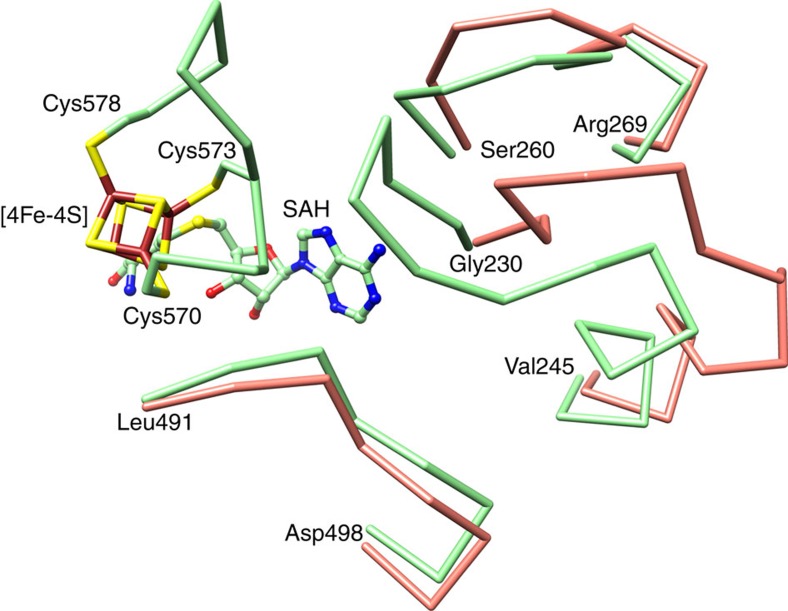
Comparison of the structure of AtThiC with bound IRN to the structure of AtThiC with bound SAH and AIR. The presence of SAH (or 5′-dAdo and L-Met) results in conformational changes for three loops near the SAM-binding site. These loops interact with the cluster-binding domain and promote docking to the active site. The largest change occurs in loop 230–245; this loop is disordered in the holo CcThiC structure. Smaller changes occur in loops 257–277 and 487–500. Ser236, which has clear electron density and forms a strong hydrogen bond with Glu241, is an outlier in the Ramachandran plot when SAH is bound, but not for the AtThiC/IRN structure.

Figure 1 was made using ChemDRAW Pro 14.0 (Perkin-Elmer, Inc.). Figs 2, 3, 4, 5, 6 and Supplementary Fig. 6 were made using Chimera^38^. Supplementary Figures showing electron density were made using PyMOL^36^.

## Author contributions

M.K.F. crystallized CcThiC and AtThiC, collected X-ray diffraction data and solved the initial structures. A.P.M. worked out conditions for preparing CcThiC and AtThiC with the [4Fe-4S] cluster, and carried out initial purifications, enzyme assays and mutant characterization. Y.Z. refined the CcThiC and AtThiC structures. S.H.A. synthesized AIR and IRN. T.P.B. directed the biochemical studies and assisted in writing the manuscript. S.E.E. directed the structural studies and wrote the manuscript, with contributions from all authors.

## Additional information

**Accession codes**: Atomic coordinates and structure factors for the reported crystal structures have been deposited with the Protein Data Bank (http://www.pdb.org/) under accession codes 4S25 (AtThiC/[4Fe-4S]/IRN/SAH/zinc trigonal crystal form), 4S26 (AtThiC/[4Fe-4S]/IRN/SAH/zinc monoclinic crystal form), 4S27 (AtThiC/[4Fe-4S]/Fe/5′-dAdo/Met/AIR), 4S28 (AtThiC/[4Fe-4S]/Fe/AIR/SAH), 4S29 (AtThiC/Fe/IRN) and 4S2A (CcThiC/[4Fe-4S]).

**How to cite this article:** Fenwick, M. K. *et al*. Non-canonical active site architecture of the radical SAM thiamin pyrimidine synthase. *Nat. Commun.* 6:6480 doi: 10.1038/ncomms7480 (2015).

## Supplementary Material

Supplementary Information Supplementary Figures 1-7, Supplementary Tables 1-2.

## Figures and Tables

**Table 1:** Data collection and refinement statistics.

	**AtThiC Fe**_**4**_**S**_**4**_**/Zn/IRN/SAH**	**AtThiC Fe**_**4**_**S**_**4**_**/Zn/IRN/SAH**	**AtThiC Fe**_**4**_**S**_**4**_**/Fe/AIR/5′-dAdo/Met**	**AtThiC Fe**_**4**_**S**_**4**_**/Fe/AIR/SAH**	**AtThiC Fe/IRN**	**CcThiC Fe**_**4**_**S**_**4**_
*Data collection*						
Space group	P3_2_21	C2	P3_2_21	P3_2_21	P3_2_21	C2
*Cell dimensions*						
*a*, *b*, *c* (Å)	107.5, 107.5, 87.6	175.9, 95.6, 71.4	106.9, 106.9, 87.6	107.1, 107.1, 87.7	107.3, 107.3, 87.8	116.7, 68.9, 97.6
*α*, *β*, *γ* (°)	90, 90, 120	90, 104.2, 90	90, 90, 120	90, 90, 120	90, 90, 120	90, 120.3, 90
Resolution (Å)	46.54–1.45 (1.50–1.45)*	48.95–1.84 (1.92–1.84)	33.89–1.27 (1.31–1.27)	40.99–1.25 (1.30–1.25)	46.46–1.38 (1.43–1.38)	45.55–2.93 (3.03–2.93)
*R*_sym_	0.06 (0.43)	0.07 (0.46)	0.04 (0.37)	0.05 (0.33)	0.05 (0.32)	0.15 (0.52)
*I*/σ*I*	13.2 (2.7)	9.9 (1.7)	15.5 (3.0)	14.5 (3.2)	16.5 (3.5)	8.0 (2.7)
Completeness (%)	97.2 (99.2)	96.9 (97.1)	99.9 (99.8)	99.0 (96.5)	98.8 (93.7)	94.2 (97.3)
Redundancy	4.8 (4.6)	2.8 (2.5)	5.3 (5.0)	5.3 (4.5)	5.4 (5.1)	2.4 (2.4)
						
*Refinement*						
Resolution (Å)	1.45	1.84	1.27	1.25	1.38	2.93
No. of reflections	100712	94814	152447	158556	118249	13613
*R*_work_/*R*_free_	0.142/0.163	0.166/0.197	0.119/0.142	0.116/0.138	0.118/0.144	0.194/0.246
*No. of atoms*						
Protein	4288	8087	4315	4336	3943	4527
Ligand/ion	66	108	68	93	43	13
Water	520	543	503	546	545	0
*B-factors*						
Protein	12.9	24.6	15.8	14.0	13.7	27.0
Ligand/ion	15.0	24.8	17.2	15.3	12.5	28.6
Water	25.7	29.1	33.8	31.5	32.6	—
*Root mean squared deviations*						
Bond lengths (Å)	0.007	0.008	0.006	0.006	0.005	0.002
Bond angles (°)	1.4	1.2	1.2	1.3	1.1	1.0

AIR, aminoimidazole ribonucleotide; AtThiC, *A. thaliana* ThiC; CcThiC, *C. crescentus* ThiC; IRN, imidazole ribonucleotide; SAH, *S*-adenosylhomocysteine; 5′-dAdo, 5′-deoxyadenosine.

^*^Highest-resolution shell is shown in parenthesis.
