# Supplementary material for: Non-canonical active site architecture of the radical SAM thiamin pyrimidine synthase
Source: Nat Commun. 2015 Mar 27;6:6480. doi: 10.1038/ncomms7480 (PMC4389238; doi:10.1038/ncomms7480)
Supplement: Supplementary Information — Supplementary Figures 1-7, Supplementary Tables 1-2. [file ncomms7480-s1.pdf]

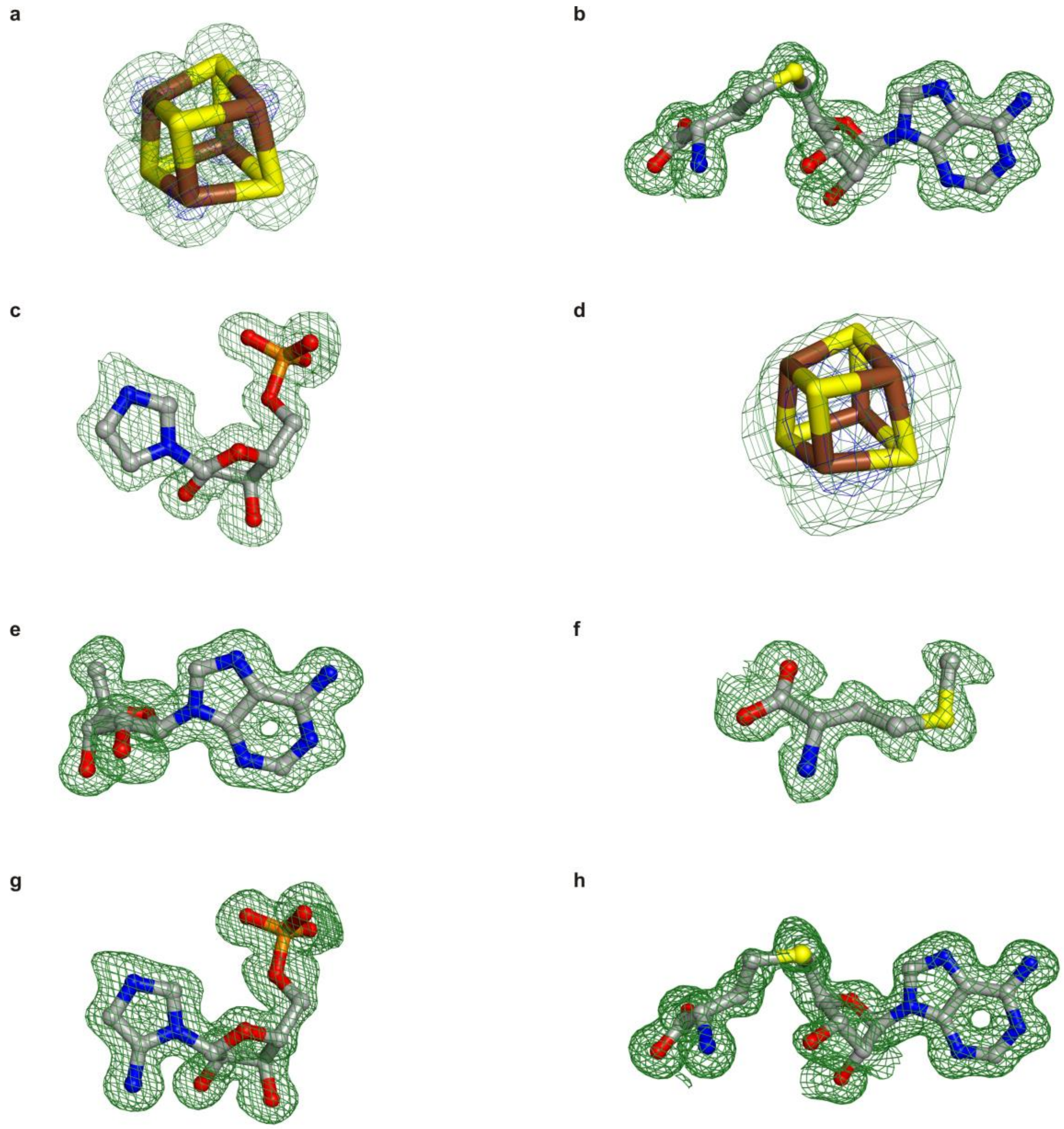

Supplementary Figure 1 | F_o-F_c electron density maps for various ligands. The figure is intended to show the quality of the electron density and provide confirmation of the identity of ligands included in the models. Each map was computed after the ligand was removed from the final refined model. Each panel is contoured at 2.5 times the RMS value of the map (green). Panel a shows a second contour level at 30 times the RMS value of the map (blue). Panel d shows a second contour level at 5 times the RMS value of the map (blue). (**a**) [4Fe-4S] cluster of AtThiC with bound SAH, IRN, and Zn. (**b**) SAH of AtThiC with bound SAH, IRN, and Zn. (**c**) IRN of AtThiC with bound SAH, IRN, and Zn. (**d**) Demonstration that the [4Fe-4S] clusters in AtThiC and CcThiC occupy the same location in the active site. A low resolution map is shown for the [4Fe-4S] cluster of CcThiC with bound SAH, IRN, and Zn. This structure was not included in Table 1 due to its low resolution; however, the location of the [4Fe-4S] cluster in the active site was clear. The structure shown is the [4Fe-4S] cluster of AtThiC after superimposition of AtThiC onto CcThiC. (**e**) 5′-dAdo of AtThiC with bound 5′-dAdo, L-Met, AIR, and Fe. (**f**) L-Met of AtThiC with bound 5′-dAdo, L-Met, AIR, and Fe. (**g**) AIR of AtThiC with bound SAH, AIR, and Fe. (**h**) SAH of AtThiC with bound SAH, AIR, and Fe.

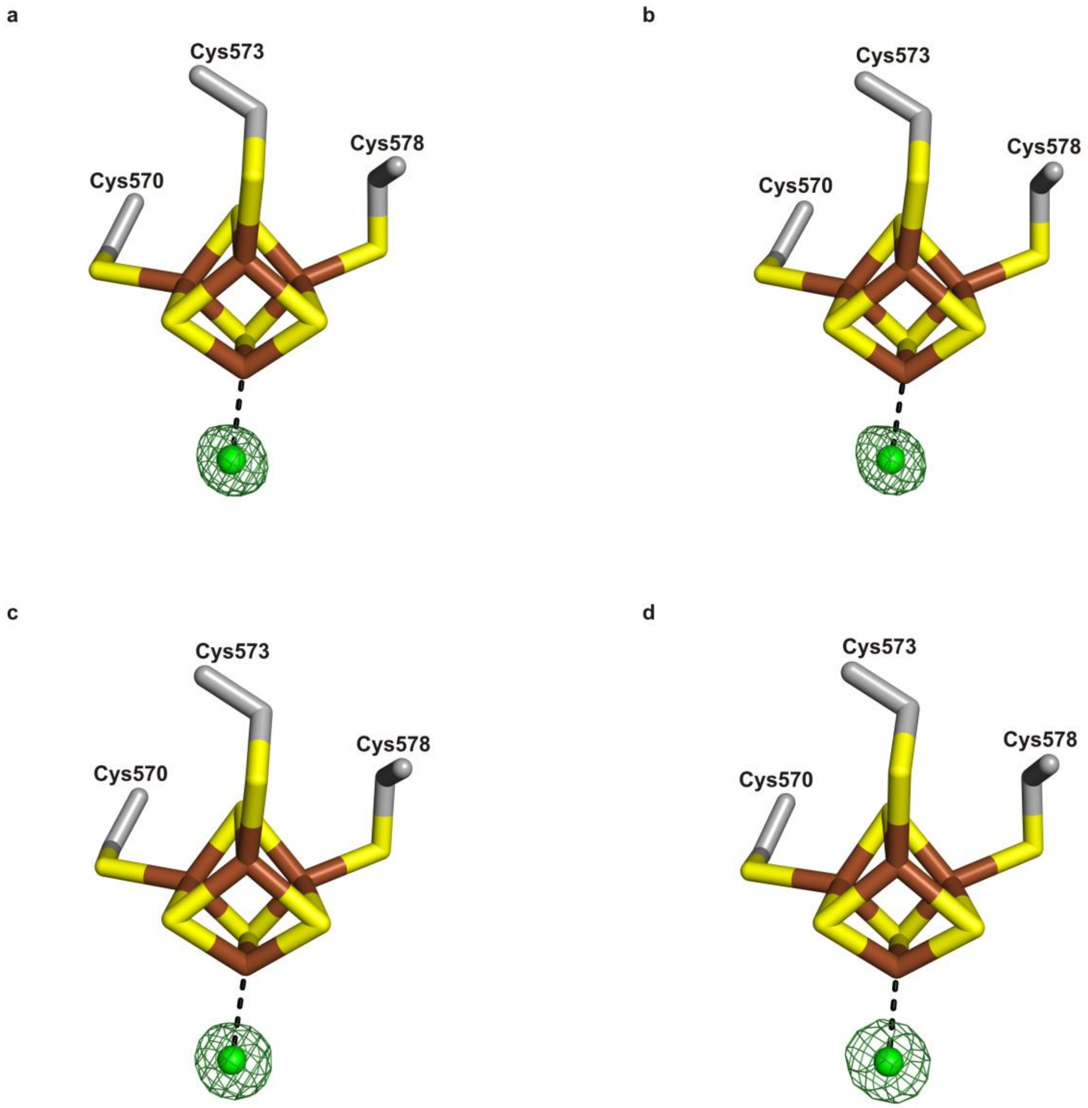

Supplementary Figure 2 | Chloride bound near differentiated Fe of the [4Fe-4S] cluster. Structures of AtThiC having the [4Fe-4S] cluster docked in the active site showed spherical electron density near the differentiated Fe of the cluster. Peak heights and B-factors were consistent with chloride. F_O - F_C electron density maps were determined for the following structures with chloride omitted: (**a**) AtThiC with bound AIR, SAH, and Fe; peak is 30 times RMS value of map, (**b**) AtThiC with bound AIR, 5′-dAdo, L-Met, and Fe; peak is 25 times RMS value of map, (**c**) AtThiC with bound IRN, SAH, and Zn (space group $P3_221$); peak is 29 times RMS value of map, and (**d**) AtThiC with bound IRN, SAH, and Zn (space group C2); peak is 18 times RMS value of map.

a

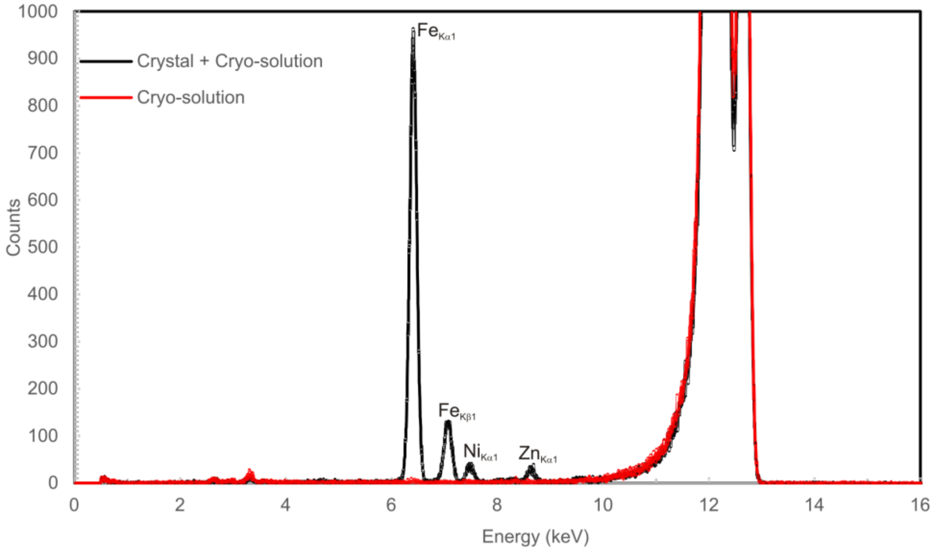

b

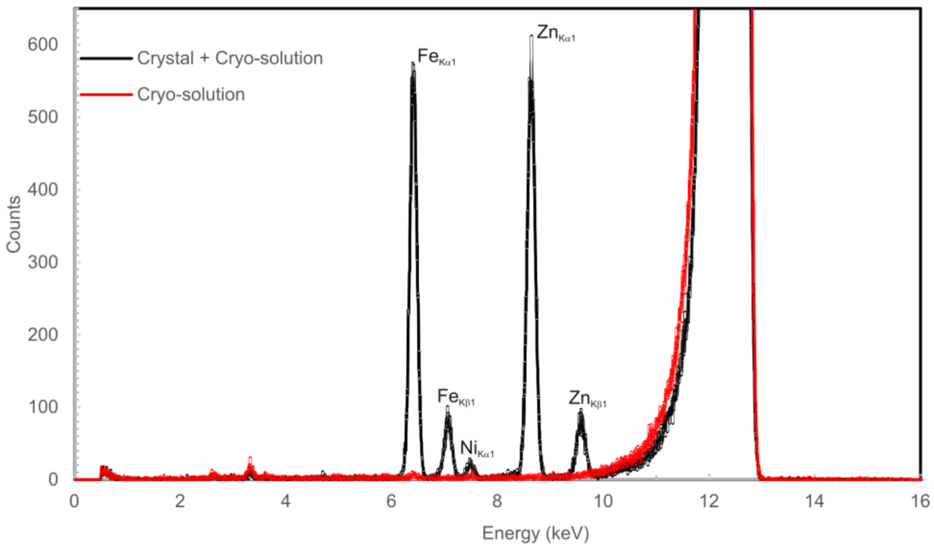

Supplementary Figure 3 | X-ray fluorescence scans on AtThiC crystals and associated cryo-solutions. (**a)** No metals added during protein purification and crystallization. (**b**) Zn added in 1:1 molar ratio to AtThiC prior to crystallization, but not added to the cryoprotectant solution. Without added Zn, strong Fe fluorescence is observed with only trace levels of Zn and Ni fluorescence. With added Zn, strong Fe and Zn fluorescence is observed plus trace levels of Ni fluorescence.

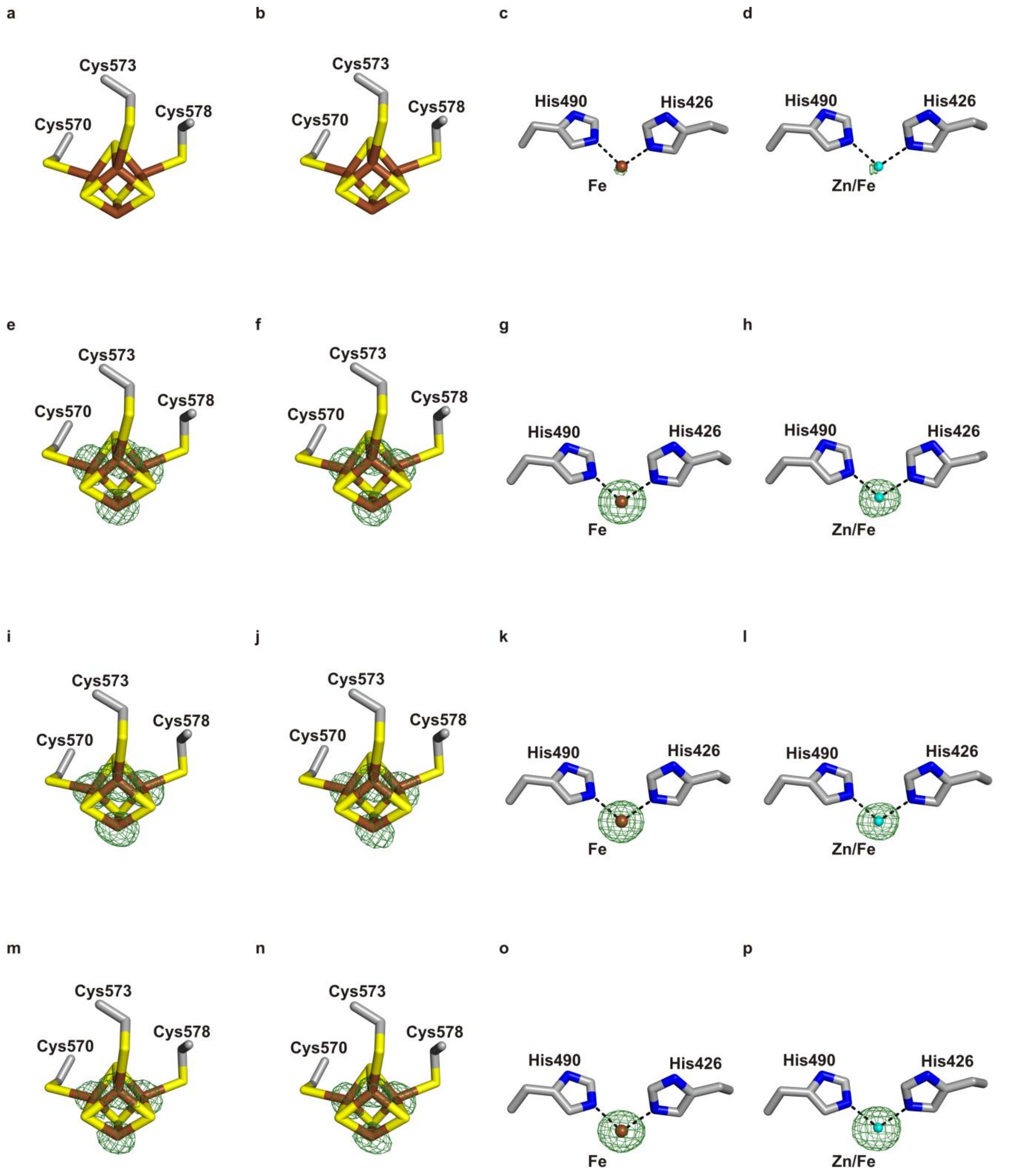

Supplementary Figure 4 | Anomalous diffraction results. Anomalous difference Fourier maps for energies (**a**-**d**) 7,100 eV (below the Fe peak energy), (**e**-**h**) 7,132 eV (at the Fe peak energy), (**i**-**l**) 9,650 eV (below the Zn peak energy), and (**m**-**p**) 9,673 eV (at the Zn peak energy). Maps are shown for the [4Fe-4S] cluster for crystals (**a**, **e**, **i**, and **m**) without Zn added and (**b**, **f**, **j**, and **n**) with Zn added, and for the secondary metal site for crystals (**c**, **g**, **k**, and **o**) without Zn added and (**d**, **h**, **l**, and **p**) with Zn added. Panels a-d show little signal because the X-ray energy is below the absorption edge of both Fe and Zn.

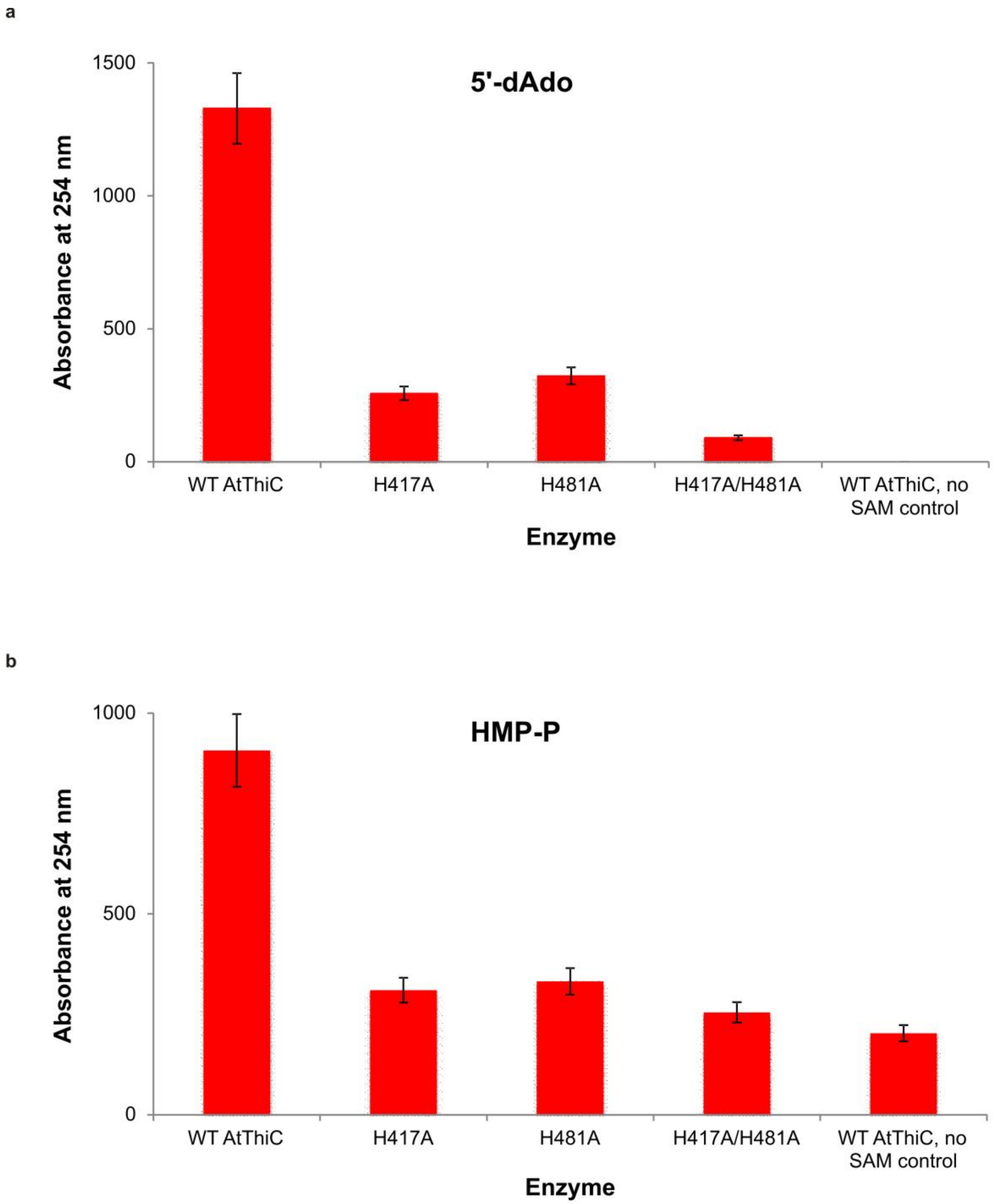

Supplementary Figure 5 | Activity of histidine mutants. The panels show HPLC peak area analysis for CcThiC and CcThiC mutants H417A, H481A, and H417A/H481A. (**a**) Levels of formation of 5′-dAdo. No SAM control indicates the amount of 5′-dAdo co-purified with the enzyme. (**b)** Levels of formation of HMP-P. No SAM control indicates the amount of HMP-P co-purified with the enzyme. The error bars represent standard deviations.

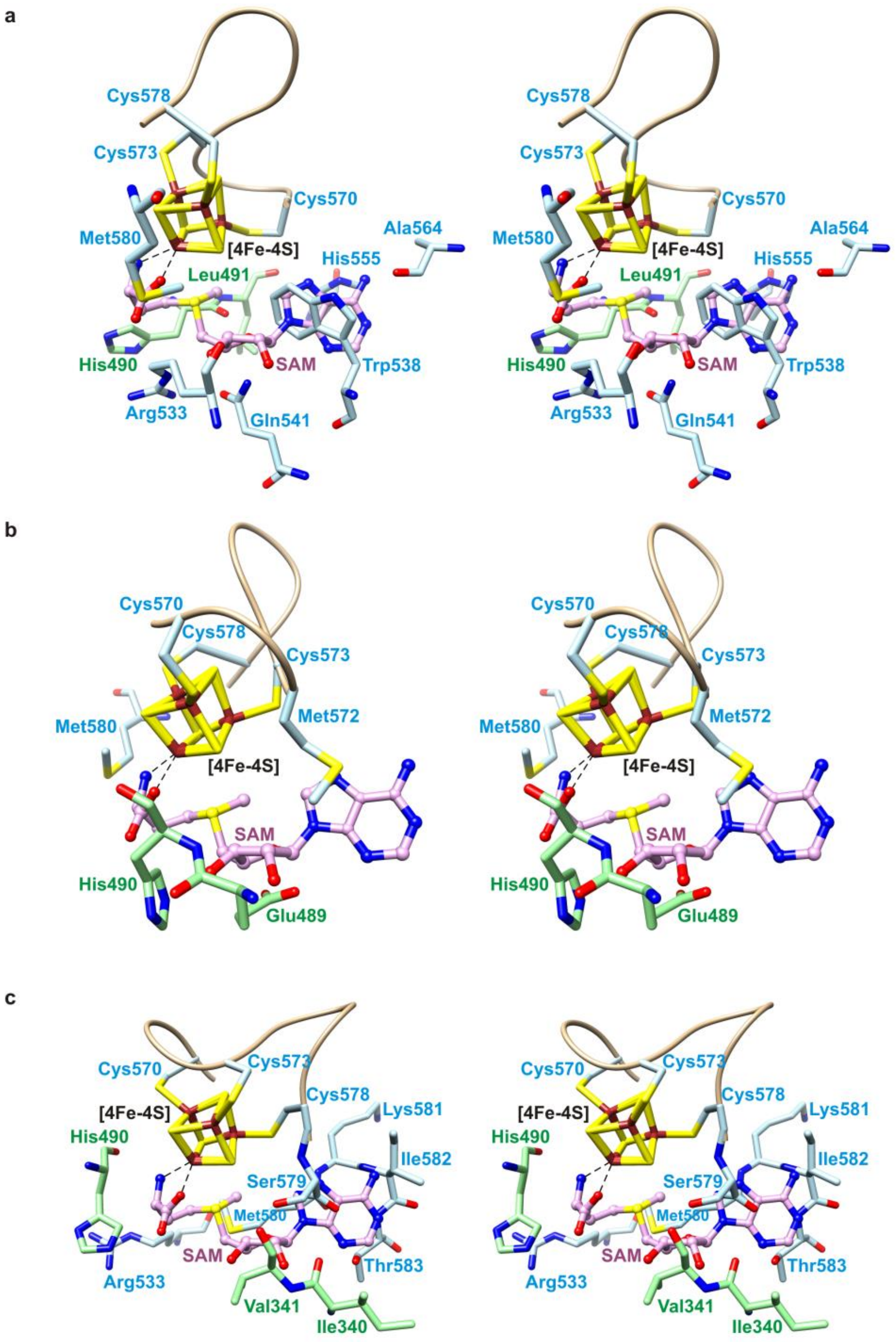

Supplementary Figure 6 | Superimposition of HydE cluster with attached SAM onto the AtThiC cluster. Assuming a canonical mode of binding of SAM to the differentiated iron of the AtThiC [4Fe-4S] cluster, three superimpositions are possible in which the SAM amino and carboxylate groups are chelated to the differentiated iron. The cluster-binding loop and residues involved in severe clashes (distances less than 2.5 Å) are illustrated with stereoviews. (**a**) Superimposition requiring the least overall movement of the cluster and SAM after first superimposing the catalytic domains of ThiC and HydE. This requires approximately a 13 Å translation and a 100° rotation of the HydE cluster. (**b**) Rotation of SAM by 120° about the cluster body diagonal containing the differentiated iron. (**c**) Rotation of SAM by 120° in the opposite direction. In addition to severe, often interpenetrating steric clashes, each model places the 5′-atom of SAM far away from the substrate AIR.

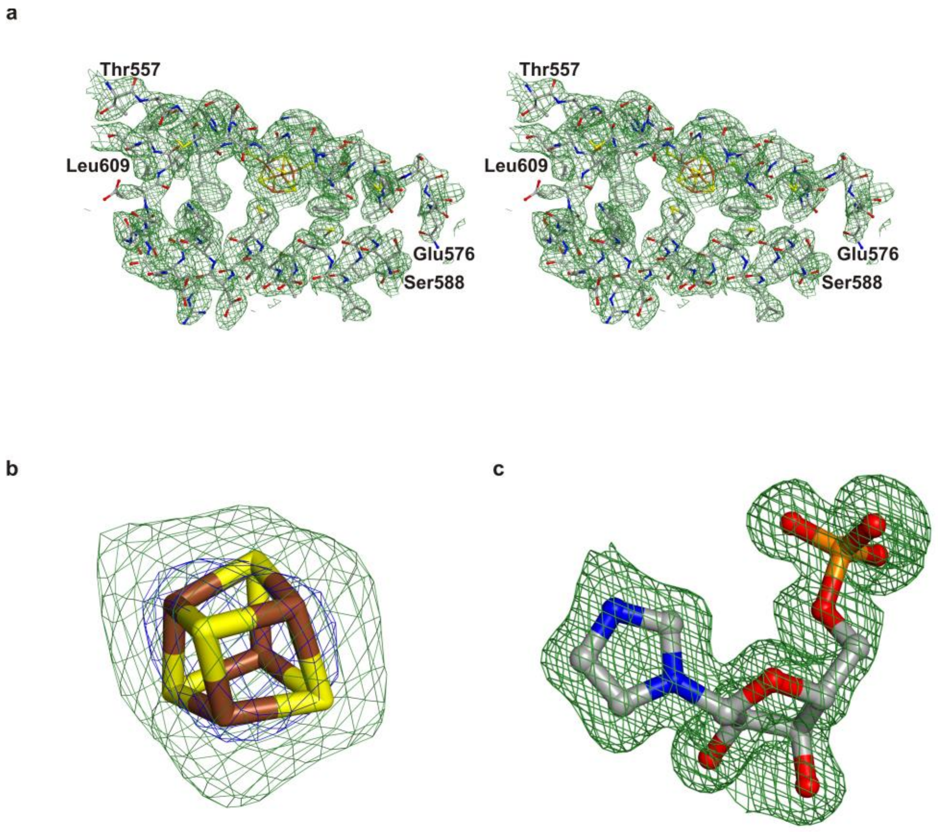

Supplementary Figure 7 | Electron density for movement of the ThiC cluster and cluster-binding domain. (**a**) Stereo image of a $2F_o$-F_c electron density map for residues 557-609 and [4Fe-4S] cluster of holo CcThiC. The map is contoured at 1 times the RMS value of the map. (**b**) F_o-F_c electron density map showing the [4Fe-4S] cluster. The cluster was omitted from the model for the calculation. The contour levels are 2.5 (green) and 9.0 (blue) times the RMS value of the map. (**c**) F_o-F_c electron density map for IRN of the AtThiC/IRN complex. IRN was omitted from the model for phase calculation. The map is contoured at 2.5 times the RMS value of the map. Even though the IRN ligand and residues of the catalytic domain are clear, electron density was not visible after residue Ser571.

Supplementary Table 1 | Analysis of multiwavelength anomalous difference Fourier maps

	Zn not added				Zn added			
	Fe peak	Fe ref	Zn peak	Zn ref	Fe peak	Fe ref	Zn peak	Zn ref
λ (eV)	7132	7100	9673	9650	7132	7100	9673	9650
f′′ Fe (e)[a]	4.0	0.6	2.4	2.4	4.0	0.6	2.4	2.4
Fe1 (e Å^{-3})[b]	0.22	0.03	0.15	0.17	0.27	0.03	0.17	0.18
Fe2 (e Å^{-3})	0.23	0.02	0.15	0.17	0.26	0.04	0.20	0.20
Fe3 (e Å^{-3})	0.21	0.02	0.16	0.17	0.25	0.02	0.19	0.19
Fe4 (e Å^{-3})	0.22	0.02	0.15	0.17	0.29	0.04	0.18	0.19
<Fe> (e Å^{-3})	0.22	0.02	0.15	0.17	0.27	0.03	0.18	0.19
exp[c]	1.00	0.15	0.60	0.60	1.00	0.15	0.60	0.60
obs[d]	0.90	0.08	0.61	0.69	1.10	0.12	0.73	0.78
f′′ Zn (e)[a]	0.9	0.9	4.0	0.6	0.9	0.9	4.0	0.6
M (RMS)	59.7	6.5	53.2	53.6	25.9	6.3	83.8	34.6
M (e Å^{-3})	0.62	0.06	0.39	0.37	0.41	0.09	0.99	0.26
obs	1.00	0.10	0.63	0.60	0.41	0.09	1.00	0.26
exp (Fe)	1.00	0.15	0.60	0.60	1.00	0.15	0.06	0.06
exp (Zn)	0.22	0.22	1.00	0.15	0.22	0.22	1.00	0.15

7,100 eV (Fe reference), 7,132 eV (Fe peak), 9,650 eV (Zn reference), and 9,673 eV (Zn peak).

[a] imaginary part of the anomalous scattering factor at the wavelength indicated
[b] peak height at the known position
[c] expected ratio of peak heights
[d] observed ratio of peak heights

Supplementary Table 2 | ThiC activity studies

conditions	% HMP-P formation	% 5′-dAdo formation
100 mM Kpi, pH 7.5, 30% glycerol	100	100
1X 1:1 Xtal:well	75	88
1.5X 1:1 Xtal:well	80	100
